# The Active Human Gut Microbiota Differs from the Total Microbiota

**DOI:** 10.1371/journal.pone.0022448

**Published:** 2011-07-28

**Authors:** Francesc Peris-Bondia, Amparo Latorre, Alejandro Artacho, Andrés Moya, Giuseppe D'Auria

**Affiliations:** 1 Joint Unit of Research in Genomics and Health, Centre for Public Health Research (CSISP) - Cavanilles Institute for Biodiversity and Evolutionary Biology (University of Valencia), Valencia, Spain; 2 Centro de Investigación en Red en Epidemiología y Salud Pública (CIBEResp), Barcelona, Spain; Charité, Campus Benjamin Franklin, Germany

## Abstract

The human gut microbiota is considered one of the most fascinating reservoirs of microbial diversity hosting between 400 to 1000 bacterial species distributed among nine phyla with *Firmicutes*, *Bacteroidetes* and *Actinobacteria* representing around 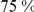 of the diversity. One of the most intriguing issues relates to understanding which microbial groups are active players in the maintenance of the microbiota homeostasis.

Here, we describe the diversity of active microbial fractions compared with the whole community from raw human fecal samples. We studied four healthy volunteers by 16S rDNA gene pyrosequencing. The fractions were obtained by cell sorting based on bacterial RNA concentration.

Bacterial families were observed to appear or disappear on applying a cell sorting method in which flow cytometry was used to evaluate the active cells by pyronin-Y staining of RNA. This method was able to detect active bacteria, indicating that the active players differed from that observed in raw fecal material. Generally, observations showed that in the active fractions, the number of reads related to *Bacteroidetes* decreased whereas several families from *Clostridiales* (*Firmicutes*) were more highly represented. Moreover, a huge number of families appeared as part of the active fraction when cell sorting was applied, indicating reads that are simply statistically hidden by the total reads.

## Introduction

The human gastrointestinal tract (GIT) hosts one of the most complex microbial communities, which has come under intensive research in recent years by applying culture-dependent and -independent methods [1]–[4]. The human gut microbiota (HGM) is a complex community in which the extremely high diversity undergoes a functional homogenization. Human gut transcriptomic related studies, highlighted as main functional roles of the gut microbiota are related to nutrient processing, energy production and synthesis of cellular components [5].

HGM is notoriously dominated by *Firmicutes* and *Bacteroidetes* phyla [2], [6], [7]. However, the functional relevance of a given taxa is not necessarily associated to its numerical dominance and taxa that represent a minority in terms of number may play important functional roles, as well as being a reservoir of key genes considering that several degradation enzymes are not coded by human genome [8]. Recent studies have shown that microbiota imbalances (dysbiosis) are associated with a wide variety of health problems. Inflammatory bowel disease, obesity, atopic syndromes, various forms of colitis, and even autism, have been linked to disruptions in human-associated microbiota, or alterations in the intimate cross-talk between microbiota and human cells [9]–[14]. Regarding obesity, Turnbaugh and collaborators observed an abnormal bloom of a specific phylum that is normally present at low concentrations in healthy individuals [15]. Also, inflammatory bowel disease, ulcerative colitis, and pouchitis have been related to changes in the gastrointestinal flora with an increase of anaerobe Gram-negative bacteria as potentially “harmful” microbiota colonizers versus the “protective” ones [16]. A general issue that deserves further investigation is to ascertain which taxa in the HGM are really metabolically active.

The study of natural microbial communities, particularly those associated to humans, is a hard task with major culture-related difficulties [17]. During the last 20 years, culture-independent identification techniques have been a big step forward in the analysis of the environmental microbial communities, as well as that associated with higher organisms. At present, the HGM is mainly studied by analyzing fecal samples, which are relatively easy to retrieve and to work with. However, there are reports of important findings showing that bacteria present in the intestinal biofilms taken from biopsy samples differ in composition from those observed in fecal ones [18]–[20].

Fecal samples are generally studied using direct approaches like 16S rDNA clone libraries or metagenomics [21]–[23]. However, the conventional DNA-based approach is unable to differentiate between viable, not viable and dead bacterial cells [24], [25]. Studies carried out on diverse types of natural samples have reported important differences in microbial composition when an RNA-based approach is applied [26]–[28]. Another feature complicating a thorough description of the HGM relates to the current lack of knowledge concerning underrepresented bacterial taxa (URB). These taxa are numerically diluted by the overwhelming presence of other taxa, which are overrepresented (ORB). This is the case, for instance, of *Firmicutes*- and *Bacteroidetes*-related phylogroups which represent about 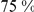 of the HGM. The remaining 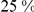 are still unknown or very poorly investigated [29], [30]. These taxa can belong to other phyla, or even to phylogroups very scarcely represented within *Firmicutes* or *Bacteroidetes*. The rare biosphere is actually a very discussed topic addressing questions about their maintenance and contribution to the whole community [31]. Different analytical tools are needed to study URB. This is the case of flow cytometry (FCM) and cell sorting techniques (CS).

Based on membrane integrity, FCM-CS has been applied to quantify viable, injured and dead bacteria from fecal samples [32]. FCM-CS have also been applied, for example, to obtain the genome scaffold of soil bacteria belonging to the TM7 phylum [33], or to derive genomic information from uncultivated marine organisms expressing a given gene [34]. Prior to the application of genomic and/or metagenomic studies, FCM-CS has also been successfully applied to count and/or enrich a given organism or population from microbial communities [35]–[39]. Finally, in order to study a selected fraction of a complex microbial community, FCM-CS techniques can be a suitable choice for culture-independent methodologies [40].

In the present work, we marked active microbial cells by means of a technique based on the presence of RNA using pyronin-Y, a fluorescent stain for total RNA. Thus, we have applied FCM-CS to sort active microbial fraction from fecal samples of four healthy volunteers. All fractions related to active and total bacteria have been characterized by 16S rDNA gene amplification and next generation sequencing (454 pyrosequencing), exploring the HGM diversity. The taxonomical distributions of ORB and URB were studied, with results indicating that in all samples active fraction differed remarkably from that obtained from raw DNA fecal samples. Several phylogroups appeared or disappeared on applying cell sorting. One of the most striking results concerns the high number of URB-related groups appearing only in the active fractions. Finally, the results suggest that the active population differs significantly from what is found by looking at the total GIT microbial population which is, up-to-date, the most common approach to gut microbiota descriptions.

## Results and Discussion

### Microbiota individual fingerprint

**Figure 1:**
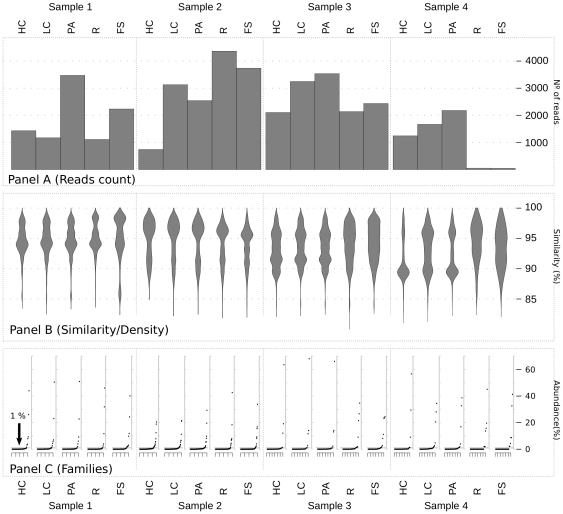
Sequencing overview. The image shows a general overview of the sequencing results. Abbreviations are defined as follow. Active fractions: PA, pyronin-Y activated; LC, low Cy5; HC, high Cy5. Total fractions: FS, Fecal Suspension; R, Ring fraction (see Methods section). Panel A reports the number of reads obtained from each sample/fraction. Panel B shows “violin plots” reporting on the X-axis the density of sequences at a given percentage of similarity (Y-axis) of the best matches from the database (see Methods section). Panel C reports the ordered percentage distribution of families for each sample/fraction; the gray dotted line labeled as “
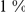
” defines the threshold used to define the two categories of URB and ORB.

Figure 1 shows a general overview of the sequencing results for each sample and fraction. Panel A shows reads count for each sample and fraction. In Panel B, the similarity values of each read with its own best match from a reference database were plotted versus the number (in percentage) of reads sharing the same value. This analysis, although quite basic, depicted a peculiar “shape” for each studied sample, which could be interpreted as a kind of fingerprint. This data reinforced our knowledge concerning the difficulties in finding patterns between individuals, when deeper taxonomic ranks (families in this case) are used.

### Global diversity report

**Figure 2:**
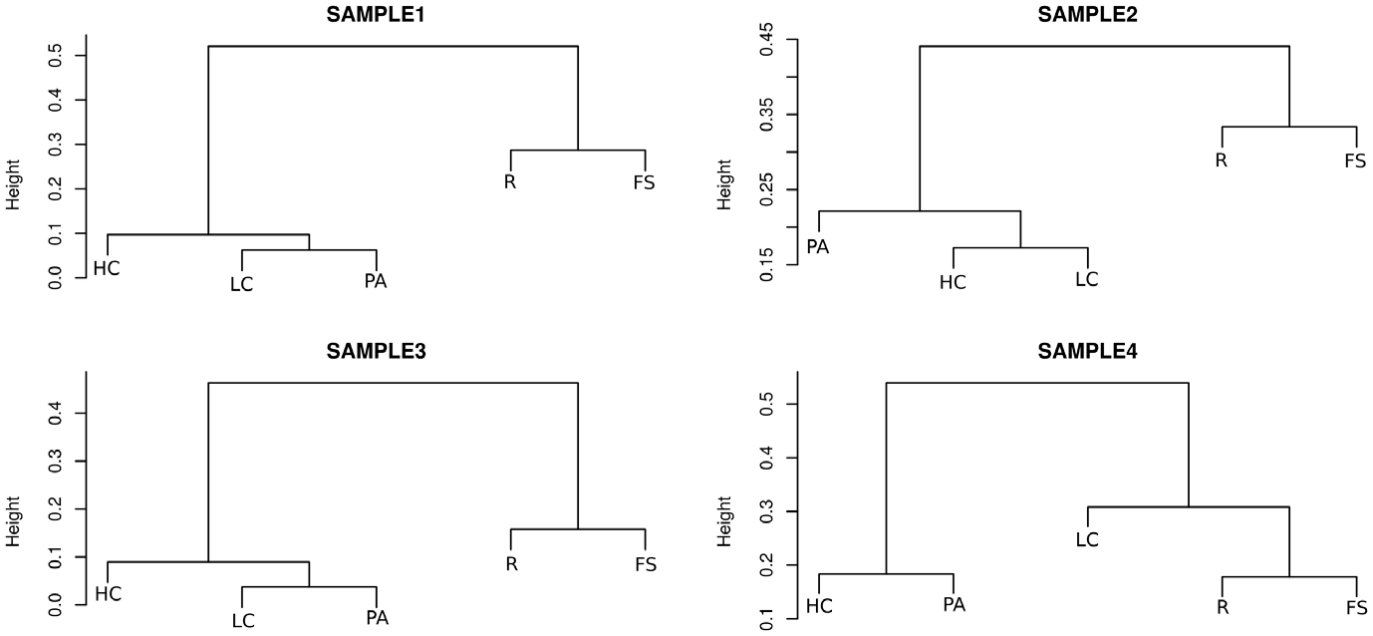
Cluster analysis among samples. Picture shows cluster analysis carried out for families table normalized to percentage. Clusters were obtained applying Bray-Curtis distance and complete agglomeration method.

**Figure 3:**
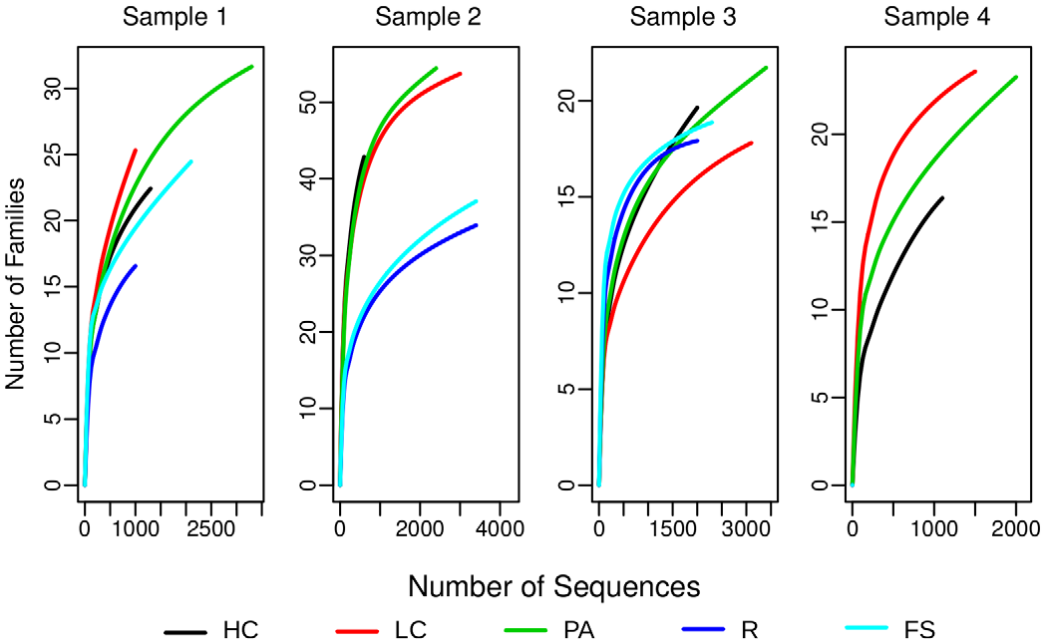
Rarefaction analysis carried out at family taxonomic rank. The X-axis shows the number of sequences in each sample/fraction, while the Y-axis shows the numbers of families encountered, respectively. Sample 4 lacks R and FS curves due to the very low number of sequences obtained from these two fractions (abbreviations as in Figure 1).

We performed a permutational multivariate analysis of variance using distance matrix (see Method section) in order to test the null hypothesis that variance between grouping factors was randomly distributed. The analysis confirmed that samples are significantly different between them as well as are active versus non-active fractions (see Table 1). Moreover, within each sample, FS and R fractions cluster together (see Figure 2). This test validated the hypothesis that active microbiota from human fecal samples is a different view of GIT microbiota and is different from what obtained from raw fecal material. This result is of particular interest considering the number of HGM descriptions which are commonly based on DNA extraction and sequencing using raw fecal material [21]–[23], [41]. Rarefaction analysis carried out at the family level, showed that curves still go up on their slopes in all samples (Figure 3). This data is further evidence of the richness in diversity of GIT microbiota [1]–[4]. Interestingly, at family level, in almost all samples we observed that curves for total fractions (FS and R, see legend of Figure 1 and methods section for abbreviations) tended to reach a plateau earlier than active fractions (HC, LC and PA). This observation clearly indicates that, with the same sequencing effort, we were able to detect more diversity of taxa in the active fraction, which are masked in FS and R fractions. All samples showed comparable dominance distributions (in terms of Shannon index, see Table S1) among samples and fractions always higher than one, which is evidence of the high richness characterized by an even distribution of many clusters comprising a few elements.

**Table 1:** Variance analysis.

SourceVar	Df	SumsOfSqs	MeanSqs	F.Model	R2	Pr( 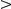 F)
ACTIVES vs TOTALS	1.00	0.31	0.31	6.66	0.17	9 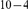 ***
SAMPLES	3.00	0.82	0.27	5.83	0.45	9 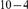 ***
FRACTIONS	3.00	0.11	0.04	0.77	0.06	0.69
Residuals	12.00	0.57	0.05		0.31	
Total	19.00	1.81			1.00	

The table shows the dependency of the diversity in relation to the three main grouping factors: active fractions versus total fractions, samples, and fractions. The variance analysis was performed with 1000 permutations. Active-versus-total and samples grouping criteria showed statistically significative differences. Columns describe: Source of variation, degrees of freedom, sequential sums of squares, mean squares, F statistics, partial R-squared and P values.

**Figure 4:**
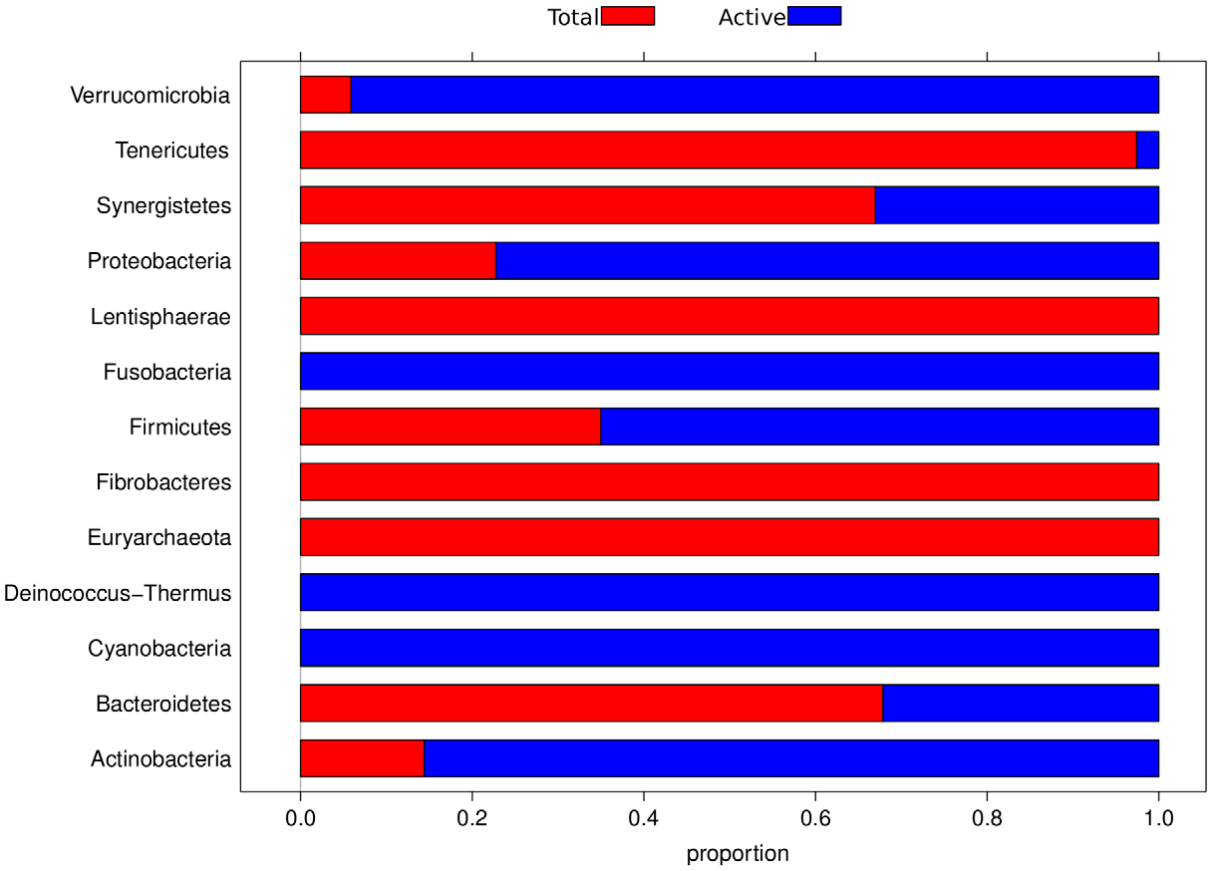
Phyla distribution. The X-axis represents the proportion of phyla from total (FS and R fraction) and active (HC, LC and PA fractions) libraries respectively (abbreviations as in Figure 1).

Figure 4 shows the phylum distributions among active and total fractions. Looking at major taxa, *Bacteroidetes* are those most represented in the total fraction, contrary to what is observed for *Firmicutes*, *Actinobacteria* and *Proteobacteria*. A more detailed view of the distribution of families is shown in Figure S1.

From all samples/fractions, 79 different families were identified belonging to 13 different phyla. As expected, the best part of the reads belonged to *Firmicutes* (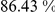), followed by *Bacteroidetes* (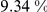), *Proteobacteria* (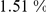), *Actinobacteria* (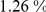), and others. Moreover, 15,735 reads out of 42,582 (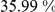) belonged to unclassified families.

**Figure 5:**
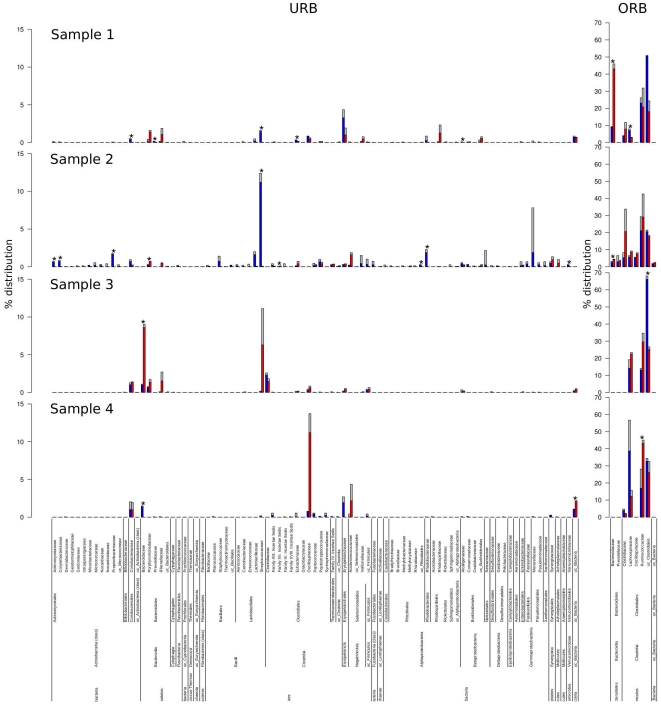
Families histogram. The histograms describe the distribution of URB and ORB families among total and active fractions. On the bottom, taxonomy ranks are reported. Bars describe percentage distribution of underrepresented (less than 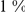, left) and overrepresented (more than 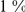 right) families. Blue and red bars describe median distribution of active (HC plus LC and PA fractions) and total (FS and R fraction) respectively. Gray bars indicate the maximum values for each family. Asterisks indicate statistically significant difference (p-value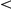 = 0.05) between active and total fractions (abbreviations as in Figure 1).

Considering all samples, 46 out of 79 families were found in total fractions (FS plus R) while up to 73 were identified from the three active fractions (HC plus LC plus PA). These results indicate that most of the families are visible only when cell sorting based on RNA content is employed. Using the inflection point identification method (see Methods section for explanation and Figures S2, S3, S4 and S5 for analytical details), we were able to divide the taxa distributions into ORB and URB phylogroups. The most recurrent inflection point was found around 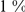 of family representativeness. Thus, we used this as the cut-off value to define ORB and URB families. Figure 5 describes the distribution of URB and ORB related families among samples and fractions. We observed that URB in total fractions were represented by 39 families (out of 46) while 65 families (out of 73) were found in the active fraction. In both cases, URB represented a considerable fraction of GIT microbial diversity, although they represented only 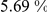 (total fractions) and the 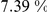 (active fractions) of reads. This data is corroborated by the high Shannon index found in all samples/fractions (see Table S1).

### Taxonomic analysis

#### *Firmicutes*

*Firmicutes* phylum comprised reads related to *Clostridia*, *Bacilli*, *Erysipelotrichi* and *Negativicutes* classes found in all samples and almost all fractions. In Sample 1, *Clostridia* were represented (on average) at 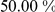 and 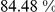 of total and active fractions respectively (with highly significant difference, see Figure 5). *Ruminococcaceae*, *Clostridiaceae* and *Lachnospiraceae* were the most commonly retrieved families from almost all samples (see Figure 5, ORB panel). Within *Clostridiales - Clostridiaceae*, reads belonging to the genus *Clostridium clariflavum* were identified in active fractions from all samples whereas they were not found in all total fractions. This organism was described as a moderately thermophilic, cellulose-/cellobiose-digesting bacteria, which was originally isolated from methanogenic sludge [42]. Other frequently recruited species, identified since 1977, were *C. irregulare*, a bile salt degrading bacteria, and *C. sporosphaeroides*, a heavy hydrogen producer (does not produce H
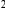
S), which is unable to convert pyruvate to butyrate, but rather, is able to convert lactate to propionate, a relatively rare, but not unique, property [43]. Another commonly shared *Clostridiaceae* species found in active fractions and in total fractions too, was *Anoxinatronum sibiricum* which was first described as an alkaliphilic saccharolytic anaerobe isolated from a natural cellulolytic community of Nizhnee Beloe (Transbaikal region) [44]. Several other *Clostridium*-related reads have been found but were not common to all samples. *Lachnospiraceae* (from ORB) were significantly the most abundant in active fractions. For instance, reads related to *Coprococcus* members (species *C. catus*, *C. comes* and *C. eutactus*) were retrieved prevalently in all active fractions from all samples; *Coprococcus* are gram-positive, anaerobic cocci active carbohydrates fermenters and producers of butyric and acetic acids with formic or propionic and/or lactic acids [45]. Other commonly recruited species from *Lachnospiraceae* were *Hespellia porcina* and *H. stercorisuis*, both able to grow on glucose, maltose, mannose, sucrose, fructose and xylose as energy sources, although the former is also able to metabolize arabinose and inositol while the latter can also grow on lactose, cellobiose, trehalose, amygdalin, and sorbitol [46]. Other common active *Lachnospiraceae* were *Roseburia faecis*, *R. hominis*, *R. inulinovorans R. intestinalis*, which are able to degrade starch with butyrate and lactate as some of the major products, and which were also described as the most active in metabolizing linoleic acid [47], [48]. Notably, butyrate has been positively associated to cancer protection [49]–[51].

*Ruminococcaceae* is the other important *Clostridiales* family in which the most common reads retrieved from active fractions were related to *Faecalibacterium prausnitzii* or *Hydrogenoanaerobacterium saccharovorans*. The former is a recognized producer of short-chain fatty acids (SCFA) with anti-inflammatory properties and was found to be reduced in Crohn disease patients [52], [53], meanwhile, the latter can ferment a number of sugars including glycogen, raffinose, sucrose, glucose, ribose, mannose, lactose, arabinose, maltose, inulin and trehalose [54]. Other common *Ruminococcaceae* were *Ruminococcus bromii*, *R. flavefaciens* and *R. gauvreauii*, which are known as normal commensal components of HGM, some of which play a protective role [55]. Also *Blautia* sp. were commonly found in all samples. Other underrepresented *Clostridiales* families were related to *Clostridiales family XI* which were found in higher abundance when associated to pathological status [56]; *Clostridiales family XII* whose genera are characterized by very heterogeneous phenotypes; *Clostridiales family XIII*, *Clostridiales family XVIII*, *Gracilibacteraceae*, *Peptostreptococcaceae*, etc.

Although we identified several families belonging to the orders *Bacillales* and *Lactobacillales*, from *Bacilli* we did not find any family common to all fractions. Finally, *Selenomonadales*, from *Negativicute*, were URB retrieved from all samples.

#### *Bacteroidetes*

In samples 1, 2 and 3, *Bacteroidaceae* were those most frequently recruited from total factions (FS and R). More specifically in Sample 1 and Sample 3 this family was represented about 9 and 5 times more (with statistical significance) in total than in active fractions. *Bacteroides dorei* and *B. uniformis* were the only two families found in all samples. Despite the high recruitment of this phylogroup in the human gut, probably its real activity is low or related to other GIT districts. It is worth mentioning that several studies indicate that *Bacteroidetes* are more abundant when a low-calorie diet is maintained [57], furthermore members of the genus *Bacteroides* are known to be short-chain fatty acid producers with a proven role against gut inflammation [53], [58]. Finally, other families retrieved in almost all samples/fractions were *Porphyromonadaceae*, *Prevotellaceae* and *Rikenellaceae* from URB.

#### *Proteobacteria*

Proteobacteria are known to have very low representation in the HGM [41]. Members of 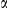-, 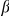-, 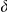
- and 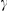 *-Proteobacteria* were found in almost all fractions as URB. Almost all families of 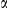 *-Proteobacteria* were more abundant in active fractions, with the exception of *Rhodospirillaceae*, *Methylocystaceae* and *Ricketsiaceae*. *Alcaligenaceae*, from 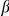 *-Proteobacteria*, were recruited in almost all samples but they were only significantly more abundant in active fractions in Sample 1. Meanwhile, 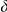 *-Proteobacteria* were identified mostly in the active fractions. *Moraxellaceae* from 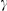 *-Proteobacteria* was the sole family recruited in all samples, although with diverse species distribution and, also in this case, from active fractions. *Pseudomonadaceae* and *Enterobacteriaceae* were found in all samples except Sample 4. Finally, for *Proteobacteria* phylum, a common profile could not be identified at a deeper taxonomic level than family, but again, despite the very low frequency of identification, these bacteria were always present as active players of HGM.

#### *Actinobacteria*

Sample 2 was the richest in *Actinobacteria* counting 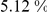, 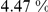 and 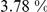 of reads for HC, LC, and PA fractions respectively; also in this case with the exception of *Coriobacteriaceae*, no family was identified as common to all samples. Members of *Actinomycetaceae*, *Coriobacteriaceae*, *Corynebacteriaceae* and *Propionibacteriaceae* were significantly more abundant in the active fractions than in the total one. Other reads belonging to *Dermabacteraceae*, *Geodermatophilaceae*, *Gordoniaceae*, *Intrasporangiaceae*, *Micrococcaceae* and *Nocardiaceae* were always found mainly in active fractions. *Actinobacteria* are reported to be underrepresented in gut phylogenetic descriptions, although there is evidence of their active role as demonstrated by FISH-related studies [30], [59]. For a detailed view on distribution of families see Figure 5.

### Conclusions

The RNA-based cell sorting approach enables the HGM total and active population to be clearly differentiated. The pyronin-Y-based sorting methodology provides more detailed information of the complex HGM community, highlighting the presence of active underrepresented bacteria hidden by the over represented ones. At family level the active fractions tend to cluster together independently of the sample. Finally, altogether the data clearly show that the functional microbiome should not be deduced uniquely from DNA-based experiments using raw fecal samples.

## Methods

### Sample collection and microbial fractions preparation

Fecal samples were obtained from four healthy human volunteers (three male and one female) between 25 and 35 years old, resident in Valencia (Spain). All volunteers follow a Mediterranean diet. The volunteers involved in this study provided their written informed consent. The study was approved by the Ethics and Research Committee of Centre for Public Health Research (CSISP) of Valencia, Spain. None of the volunteers had intestinal organic disorders or recent treatment with antibiotics. Samples were collected in sterile 30 ml screw-cap containers (25×90 mm; PP SPOON; DELTALAB), containing 8 ml RNAlater (Ambion #AM7020) in order to preserve RNA. The samples were delivered to the laboratory within 24 h and stored at 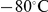. For each sample, around one gram of fecal material was suspended by vortexing (2 min). Fecal suspension was centrifuged (800 g) for 2 min to pellet big aggregates. Supernatant was centrifuged at 7500 g for 7 min to collect microbial cells from fecal suspension. Pellet was washed twice in PBS (Phosphate Buffer Saline, Sigma-Aldrich #P4417-100TAB). A sub-sample of fecal suspension (hereinafter “FS”) was stored, considered as the total microbiota control for each sample in order to describe the whole fecal microbial community (standard metagenomic approach). Two concentrations of Histodenz (Sigma-Aldrich #D2158), (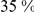 and 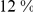), were dissolved in PBS and sterilized by 0.22 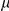
m filtration; 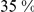 and 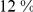 Histodenz solutions were stratified (2 ml each) in 15 ml sterile centrifuge tubes. Two milliliters of fecal suspension were finally deposited onto the 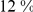 Histodenz layer and centrifuged for 7 min at 5000 g in a swing out rotor centrifuge at 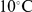. This step is crucial for detached microbial cells collection. Time, speed and temperature were optimized to avoid over-centrifugation, which could produce aggregation of microbial cells (data not shown). After centrifugation, two stratifications could clearly be identified on top of the 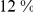 and 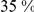 layers and a pellet at the bottom of the tube (see Figure S6). Upper PBS and 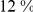 layers were gently removed by pipetting. Cells floating on the 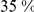 layers (called “Ring fraction”, hereinafter “R” fraction), containing microbial cells, were aspired and moved into a sterile 1.5 ml tube, washed twice and resuspended finally in 900 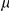
l of PBS. Microscopical observation of 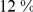 layer showed few bacterial cells as well as some debris; pellet at the bottom of the tube showed mainly fibers and microbial aggregates. Cells were immediately fixed adding 100 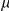
l of 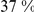 formaldehyde (final concentration: 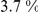) and incubated over-night at 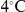. Fixed cells were washed twice to remove residual formaldehyde.

#### Fluorescent *in situ* hybridization (FISH)

A subsample of 100 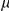
l of cells from R fraction, with an optical density (O.D. 600) around 1, were permeabilized by lysozyme (Sigma-Aldrich #L7651 approx 120 units/mg protein, 9 min at 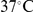) in order to facilitate probe diffusion into Gram-positive bacteria. Afterwards, tubes were chilled on ice and washed twice in PBS. Microbial cells from the previous step were resuspended in 50 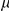
l of hybridization buffer (final concentration: NaCl 0.9 M, TrisHCl 0.02 M, SDS 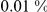). One 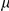
l (0.5 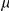
g/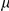
l) of each Cy5-labeled probe was added to the mixture (see Table S2 for probe details). Hybridization was carried out for three hours at 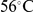. In order to remove non-specifically hybridized probes, 1 ml of hybridization buffer was added to the tubes and transferred to a 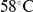 bath for 15 min. Hybridization mixture was then washed twice in PBS and finally resuspended in 1 ml of PBS. The attempt to distinguish between highly represented phylogroups and the rest of the bacteria, supposedly not hybridized to fluorescent probes, did not work as expected, resulting in an undifferentiated distribution of reads between HC and LC samples (data not shown). A further optimization is required.

#### RNA staining

One microliter of pyronin-Y (Sigma-Aldrich #P9172, 10 mg/ml) was added to the sample from previous step (1 ml of volume) for total RNA staining and incubated for 20 min at 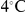. Cells were then collected by centrifugation and washed twice in PBS and finally resuspended in 50 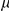
l of PBS. Cells were stored at 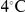 before sorting (same day).

#### Cell Sorting (CS)

Microbial cell sorting was carried out using the MoFloTM XDP cell sorter. The cytometer emission filter was the 580/30. The trigger was in FL2. The light sources were the Argon 488 nm (blue) laser (200 mW power) and the 635 nm (red) diode laser (25 mW power). The lasers were aligned using Flow-Check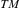 (10
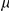
m) and Flow-Set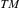 (3
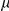
m) Beckman Coulter Beads.

**Figure 6:**
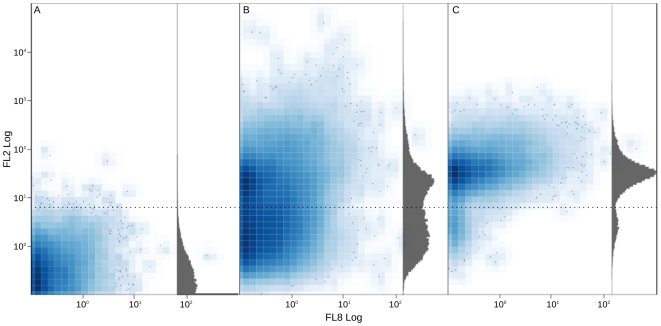
Cytometry dotplot. The three panels show the distribution of events in FL8 PMT versus FL2 PMT. X and Y axis are defined in logarithmic arbitrary units. Gray histograms resume the distribution of events along FL2 PMT. Panel A shows unstained cells. Panel B shows cells stained with pyronin-Y with trigger in SS PMT; it is possible to appreciate the two populations of unstained and stained cells respectively (dotted black line separates the two populations). Panel C shows only pyronin-Y stained cells passing the threshold on FL2 PMT.

One microliter of each sample was diluted in 10 ml of 0.2 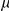
m filtered PBS. FL2 PMT (Photo Multiplier Tube) detected fluorescence emitted by excited pyronin-Y. Control experiments to set the discriminator level were carried out with samples not stained with pyronin-Y (see Figure 6). All particles not stained with pyronin-Y (spore, inactive or dead cells and debris) were discriminated and filtered out. Thus, we restricted the analysis to cells with high RNA concentration only; this fraction was called “PA” (pyronin-Y activated, Figure S7, green frame). Number of cells sorted from each fraction are reported in Table 2. Moreover we attempted to separate cells by Cy5 fluorescent probe, obtaining not-hybridized (“LC”, low Cy5, dark green frame) and Cy5-hybridized (“HC”, high Cy5, red frame) cell fractions. All fractions were sorted into sterile plastic tubes for further applications (see Figure S7 for a general view of FCM-CS dotplots). A schema clarifying the methodology is provided in Figure S8.

**Table 2:** Number of cell sorted for fraction.

Sample	Fraction	Number of cells
SAMPLE1	PA	366.637
	LC	55.440
	HC	363.219
SAMPLE2	PA	38.555.405
	LC	2.732.462
	HC	30.272.597
SAMPLE3	PA	29.773.688
	LC	3.404.025
	HC	13.391.699
SAMPLE4	PA	6.834.332
	LC	2.685.887
	HC	2.952.656

The table shows the number of cells collected by cell sorter for each sample and fraction.

### DNA extraction and rDNA amplification

DNA extraction from all fractions was carried out using the CTAB method [60]. Total 16S rDNA was amplified from each fraction using 27F and 530R universal primers [61]. Forward and reverse primers were linked to multiplex identifier (MID) and “PERMUTAG” (WGNGNNGW), a combinatory tag to discriminate between exactly equal sequences coming from a documented pyrosequencing bias related to emulsion PCR [62], from which are fully identical sequences. PERMUTAGs allow 256 possible combinations, permitting discrimination of PCR products originated from different templates. The structure for each primer was finally MID-PERMUTAG-TC(linker)-Primer (see Table S3 for details). PCR products obtained from each fraction/sample were purified by Nucleofast 96 PCR filter plates (Macherey Nagel #74310050), pooled together in equimolar concentration and sequenced by massive parallel 454 pyrosequencing (Roche) in 1/8 of plate (Titanium chemistry).

### Bioinformatics and statistic analyses

Each fraction was initially dereplicated using CDHIT software [63] in order to remove those reads with the same length and sequence, considering the PERMUTAG as part of the sequence. MIDs and PERMUTAGs were finally removed leaving a dataset of 42,582 clean reads with an average length of 492.97 nt. The phylogenetic assignment was carried out by software developed *ad-hoc* in our laboratory based on the last common ancestor (LCA) algorithm (available at https://github.com/emepyc/Blast2lca).

Descriptive and statistical analyses were carried out with the R statistic package [64].

The analysis of variance (Table 1) was carried out on normalized taxonomy table having samples in rows and families in columns. Bray-Curtis distances were calculated. The obtained matrix was then used to perform the analysis of variance following various grouping criteria. “ACTIVES vs TOTALS” shows the analysis of variance grouping data by active fractions (PA, HC and LC) versus total fractions (FS and R). “SAMPLES” line describes the significance of variance grouping the taxonomy matrix by sample. “FRACTIONS” describes variance among the five fractions. Stars indicates highly significant values. The analysis was performed by Adonis method from Vegan R package [64], [65] with 1000 permutations.

A mathematical approach was employed to define if a given family belonged to the URB or ORB fraction respectively. To do so, for each sample/fraction, the list of family abundance values was sorted and the value corresponding to the last inflection point was used as cut-off. The reads belonging to active (HC, LC and PA) and total fractions (R and FS) were respectively summarized and median values were used to perform the Welch Two Sample t-test which is an adaptation of the Student's t-test intended for use with two samples having possibly unequal variances.

Flow cytometry data were analyzed with R package flowCore and flowViz from Bioconductor [64], [66]–[68].

### Accession numbers

Sequences were deposited in NCBI Sequence Read Archive (SRA) database (SRP005393).

## Supporting Information

Figure S1 **Clustering analysis.** The figure shows the heatmap built on abundance values of families (in percentage) for each sample/fraction. Legend describes percentage ranges; blue gradient goes exponentially from 0.0001 to 1 representing URB distributions, brown gradient representing ORB. Dendrogram on top of the chart clusters the fractions. Abbreviations are defined as follow. Active fractions: PA, pyronin-Y activated; LC, low Cy5; HC, high Cy5. Total fractions: FS, Fecal Suspension; R, Ring fraction (see Methods section).
(PDF)

Figure S2 **Inflection points. Sample 1.** Top panels show the second derivative used to calculate inflection points for each fraction. Bottom panels show ordered family distributions in percentages. Red lines describe the smoothed curve calculated for ordered family distribution data points. Dashed vertical lines mark the identified inflection points for each fraction (continue…).
(PDF)

Figure S3 **Inflection points. Sample 2.** (Follow): Top panels show the second derivative used to calculate inflection points for each fraction. Bottom panels show ordered family distributions in percentages. Red lines describe the smoothed curve calculated for ordered family distribution data points. Dashed vertical lines mark the identified inflection points for each fraction (continue…).
(PDF)

Figure S4 **Inflection points. Sample 3.** (Follow): Top panels show the second derivative used to calculate inflection points for each fraction. Bottom panels show ordered family distributions in percentages. Red lines describe the smoothed curve calculated for ordered family distribution data points. Dashed vertical lines mark the identified inflection points for each fraction (continue…).
(PDF)

Figure S5 **Inflection points. Sample 4.** (Follow): Top panels show the second derivative used to calculate inflection points for each fraction. Bottom panels show ordered family distributions in percentages. Red lines describe the smoothed curve calculated for ordered family distribution data points. Dashed vertical lines mark the identified inflection points for each fraction.
(PDF)

Figure S6
**Microbial cell preparation from fecal samples.** Microscopy photograph on the left (panel a) shows DAPI stained microbial cells obtained from R fraction recovered from 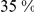 Hystodenz layer (panel b). Photograph on the right (panel c) shows DAPI stained microbial cells from pellet layer with several fiber-like structures and microbe aggregates.(PDF)

Figure S7 **Cytometry dotplot.** Fluorescence dotplot of pyronine-Y-activated cells. The X-axis describes the intensity of fluorescence emitted by each cell (arbitrary units), measured on the FL8 photomultiplier. The Y-axis describes the intensity of the fluorescence emitted by each cell passing over the FL2 discriminator (bacteria stained with pyronin-Y). The PA region was used to collect all pyronin-Y activated cells; LC region collected cells with low or null Cy5 fluorescence emission; HC region collected mainly cells hybridized with group-specific probes with high Cy5 fluorescence emission. Flow cytometry data were analyzed with R package flowCore and flowViz by Bioconductor [64], [66]–[68].
(PDF)

Figure S8 **Protocol schema.** Arrows define the work flow. Black arrows ideally represents all cells and particles contained in the samples. Red arrows represents the fraction of the microbiota hybridized to CY5 probes. Green arrows represents the fraction of cells labeled with pyronin-Y. Double colored arrows indicate cells stained simultaneously with pyronin-Y and CY5 fluorescent probes. Gray arrows represents the unstained fraction (supposedly inactive, spore, dead cells or simply debris). In bold are represented the fractions obtained for downstream sequencing.
(PDF)

Table S1 **Diversity indexes. Main diversity indexes calculated at family taxonomy rank for each sample/fraction.**
(PDF)

Table S2 **Probes used in this work** [**69]**–[73****] **.**
(PDF)

Table S3 **Multiplex Identifiers (MIDs) list and universal 16S rRNA primers used in this work** [**61**] **.**
(PDF)
