# Supplementary figures and images for: The Active Human Gut Microbiota Differs from the Total Microbiota
Source: PLoS One. 2011 Jul 28;6(7):e22448. doi: 10.1371/journal.pone.0022448 (PMC3145646; doi:10.1371/journal.pone.0022448)

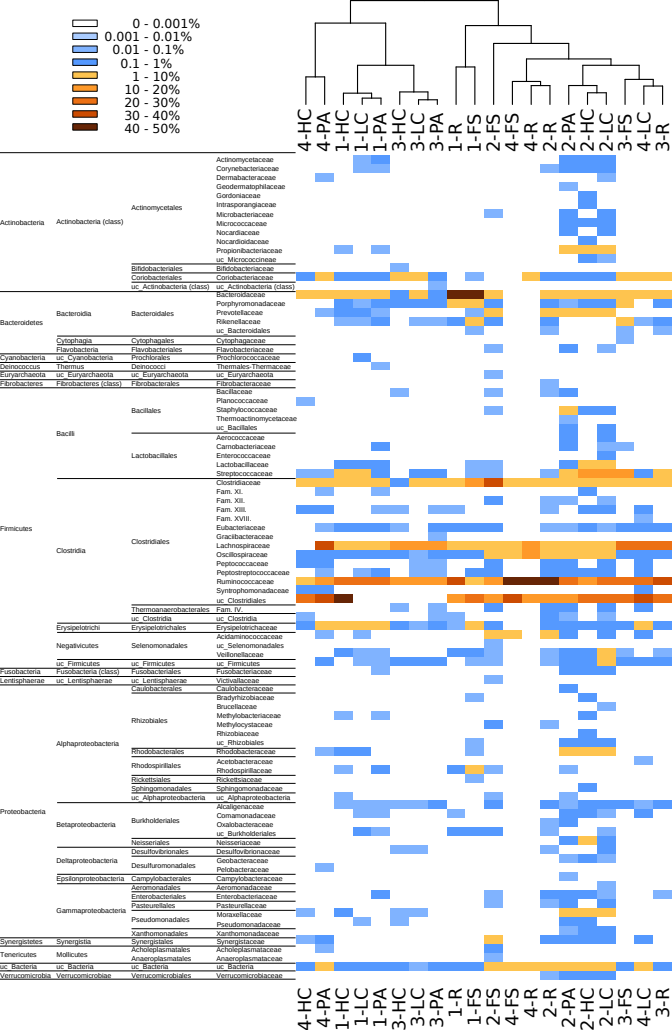

Supplement: Figure S1 — Clustering analysis. The figure shows the heatmap built on abundance values of families (in percentage) for each sample/fraction. Legend describes percentage ranges; blue gradient goes exponentially from 0.0001 to 1 representing URB distributions, brown gradient representing ORB. Dendrogram on top of the chart clusters the fractions. Abbreviations are defined as follow. Active fractions: PA, pyronin-Y activated; LC, low Cy5; HC, high Cy5. Total fractions: FS, Fecal Suspension; R, Ring fraction (see Methods section). (PDF) [file pone.0022448.s001.pdf]

# Sample 1

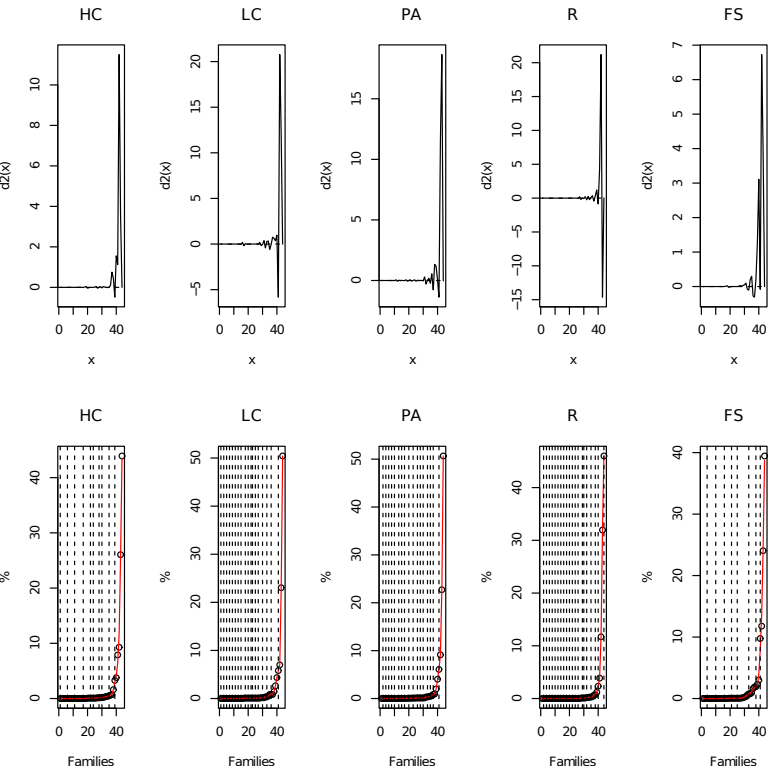

Supplement: Figure S2 — Inflection points. Sample 1. Top panels show the second derivative used to calculate inflection points for each fraction. Bottom panels show ordered family distributions in percentages. Red lines describe the smoothed curve calculated for ordered family distribution data points. Dashed vertical lines mark the identified inflection points for each fraction (continue…). (PDF) [file pone.0022448.s002.pdf]

# Sample 2

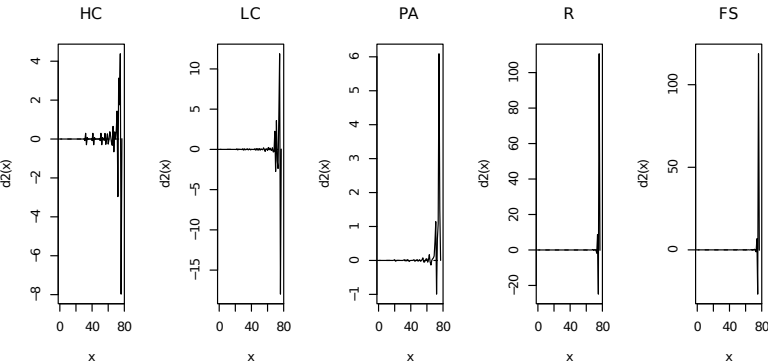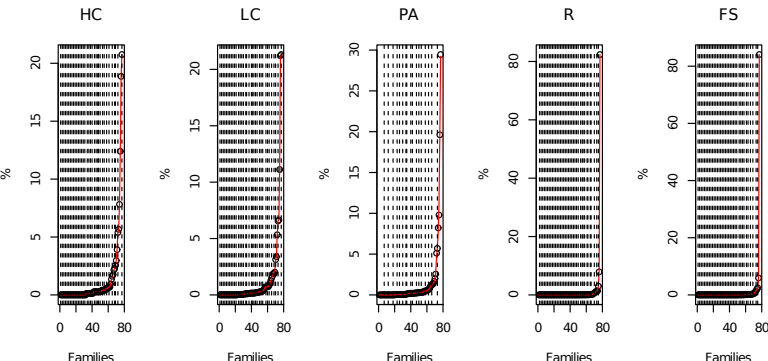

Supplement: Figure S3 — Inflection points. Sample 2. (Follow): Top panels show the second derivative used to calculate inflection points for each fraction. Bottom panels show ordered family distributions in percentages. Red lines describe the smoothed curve calculated for ordered family distribution data points. Dashed vertical lines mark the identified inflection points for each fraction (continue…). (PDF) [file pone.0022448.s003.pdf]

# Sample 3

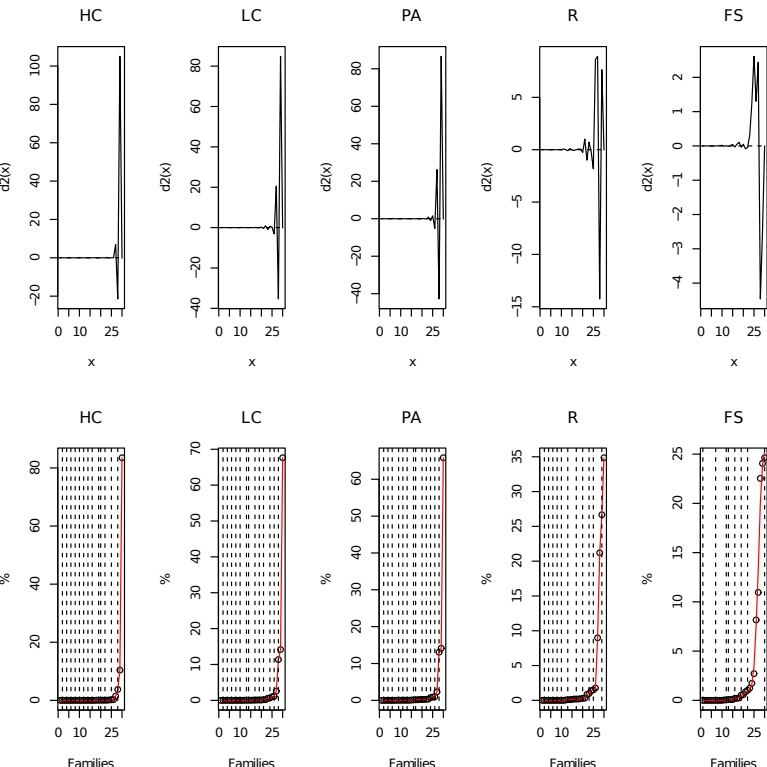

Supplement: Figure S4 — Inflection points. Sample 3. (Follow): Top panels show the second derivative used to calculate inflection points for each fraction. Bottom panels show ordered family distributions in percentages. Red lines describe the smoothed curve calculated for ordered family distribution data points. Dashed vertical lines mark the identified inflection points for each fraction (continue…). (PDF) [file pone.0022448.s004.pdf]

# Sample 4

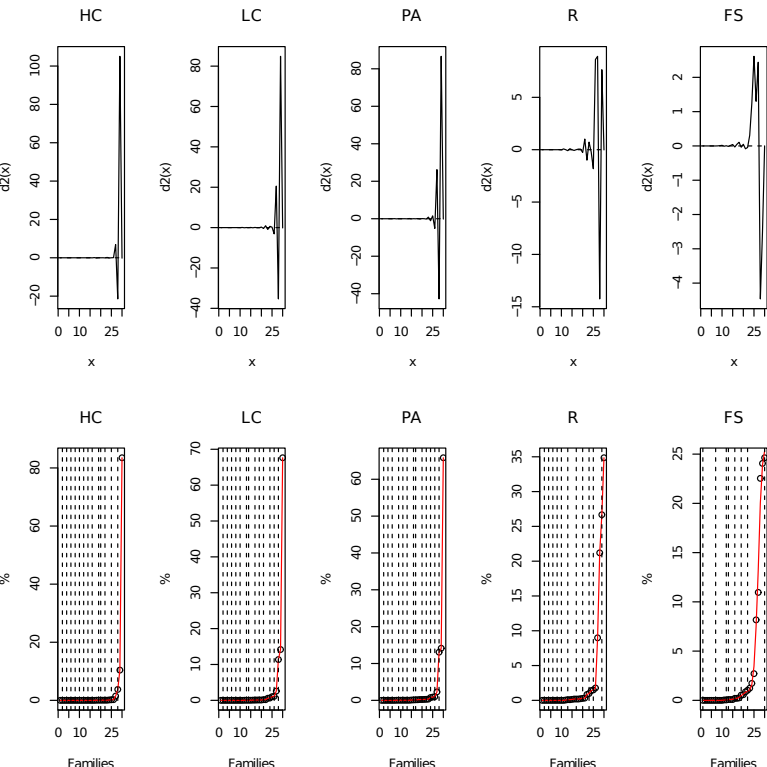

Supplement: Figure S5 — Inflection points. Sample 4. (Follow): Top panels show the second derivative used to calculate inflection points for each fraction. Bottom panels show ordered family distributions in percentages. Red lines describe the smoothed curve calculated for ordered family distribution data points. Dashed vertical lines mark the identified inflection points for each fraction. (PDF) [file pone.0022448.s005.pdf]

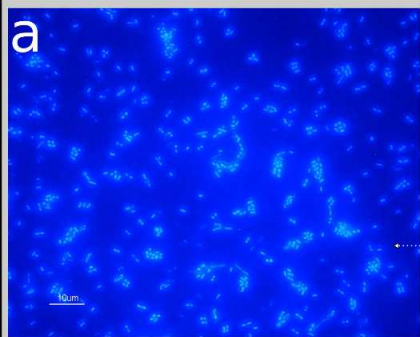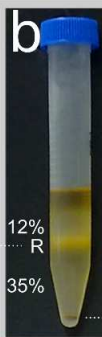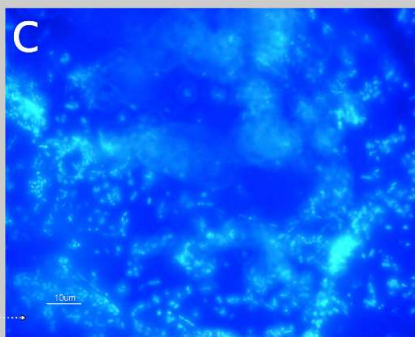

Supplement: Figure S6 — Microbial cell preparation from fecal samples. Microscopy photograph on the left (panel a) shows DAPI stained microbial cells obtained from R fraction recovered from Hystodenz layer (panel b). Photograph on the right (panel c) shows DAPI stained microbial cells from pellet layer with several fiber-like structures and microbe aggregates. (PDF) [file pone.0022448.s006.pdf]

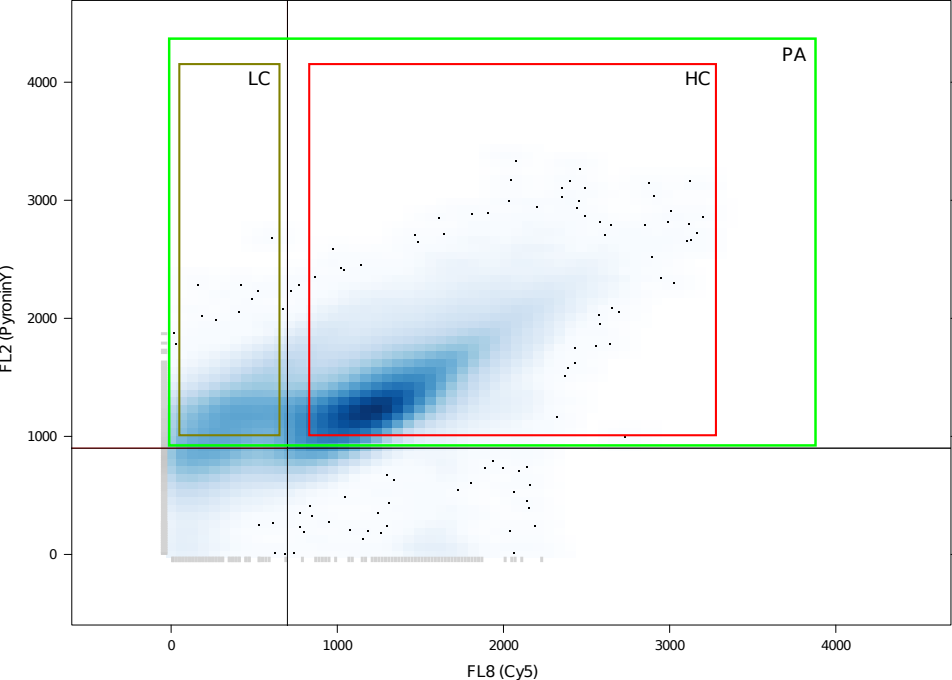

Supplement: Figure S7 — Cytometry dotplot. Fluorescence dotplot of pyronine-Y-activated cells. The X-axis describes the intensity of fluorescence emitted by each cell (arbitrary units), measured on the FL8 photomultiplier. The Y-axis describes the intensity of the fluorescence emitted by each cell passing over the FL2 discriminator (bacteria stained with pyronin-Y). The PA region was used to collect all pyronin-Y activated cells; LC region collected cells with low or null Cy5 fluorescence emission; HC region collected mainly cells hybridized with group-specific probes with high Cy5 fluorescence emission. Flow cytometry data were analyzed with R package flowCore and flowViz by Bioconductor [64], [66]–[68]. (PDF) [file pone.0022448.s007.pdf]
