# Supplementary material for: The Active Human Gut Microbiota Differs from the Total Microbiota
Source: PLoS One. 2011 Jul 28;6(7):e22448. doi: 10.1371/journal.pone.0022448 (PMC3145646; doi:10.1371/journal.pone.0022448)
Supplement: Figure S8 — Protocol schema. Arrows define the work flow. Black arrows ideally represents all cells and particles contained in the samples. Red arrows represents the fraction of the microbiota hybridized to CY5 probes. Green arrows represents the fraction of cells labeled with pyronin-Y. Double colored arrows indicate cells stained simultaneously with pyronin-Y and CY5 fluorescent probes. Gray arrows represents the unstained fraction (supposedly inactive, spore, dead cells or simply debris). In bold are represented the fractions obtained for downstream sequencing. (PDF) [file pone.0022448.s008.pdf]

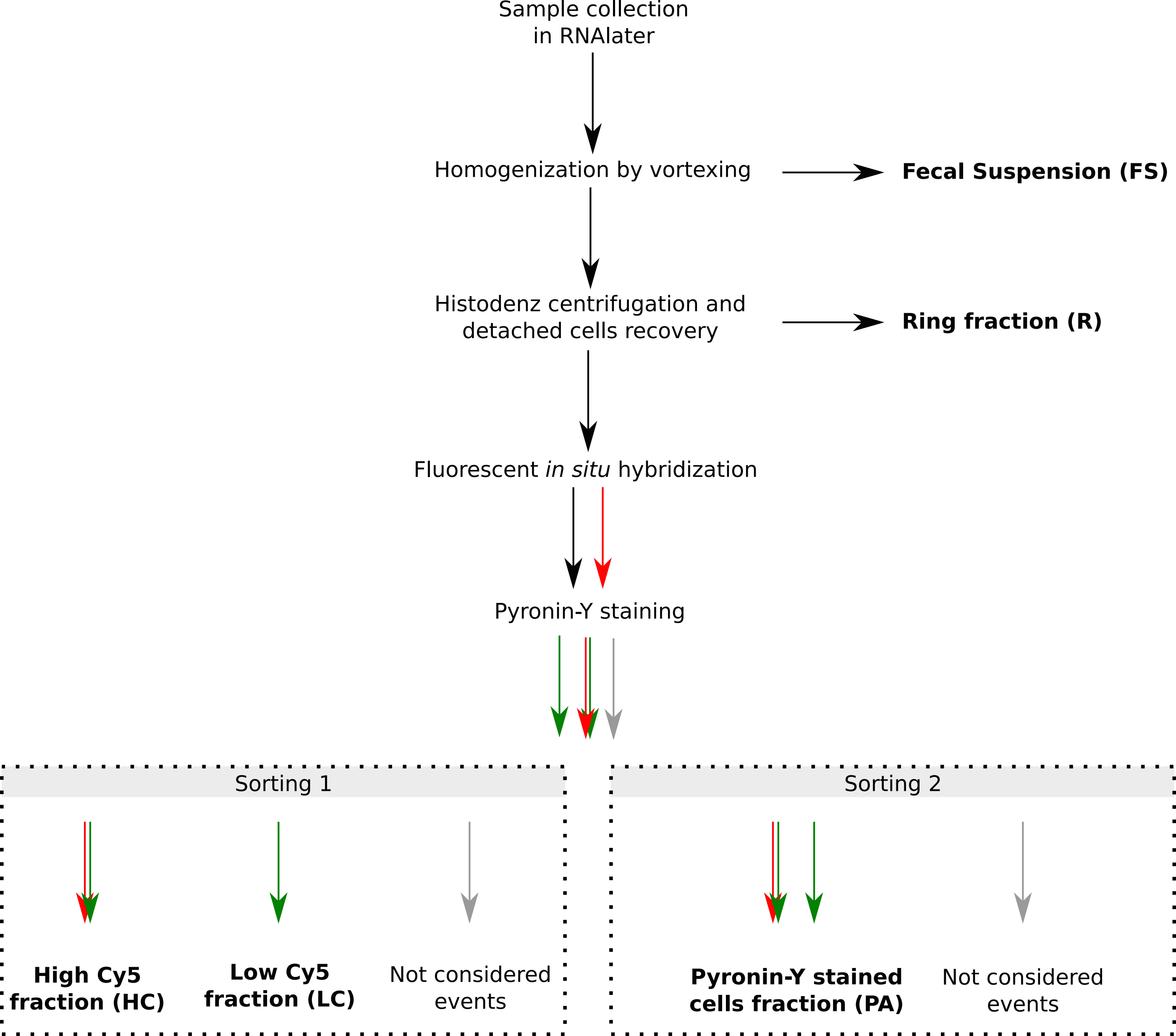
Sample collection
in RNAlater
Homogenization by vortexing
Fecal Suspension (FS)
Histodenz centrifugation and
detached cells recovery
Ring fraction (R)
Fluorescent in situ hybridization
Pyronin-Y staining
Sorting 1
High Cy5
fraction (HC)
Low Cy5
fraction (LC)
Not considered
events
Sorting 2
Pyronin-Y stained
cells fraction (PA)
Not considered
events
