# Supplementary material for: The Active Human Gut Microbiota Differs from the Total Microbiota
Source: PLoS One. 2011 Jul 28;6(7):e22448. doi: 10.1371/journal.pone.0022448 (PMC3145646; doi:10.1371/journal.pone.0022448)
Supplement: Table S1 — Diversity indexes. Main diversity indexes calculated at family taxonomy rank for each sample/fraction. (PDF) [file pone.0022448.s009.pdf]

Sample	Fraction	N	Shannon	Chao1	SE.Chao1	ACE	SE.ACE
1	HC	23	1.66	28.00	10.17	28.77	2.73
	LC	27	1.62	40.75	17.42	47.50	3.93
	PA	32	1.56	37.25	8.28	35.62	2.73
	R	17	1.41	18.50	3.49	21.07	2.27
	FS	25	1.78	43.00	49.09	47.77	3.43
2	HC	45	2.69	48.27	3.60	50.03	3.36
	LC	54	2.66	61.00	13.15	57.41	3.69
	PA	55	2.50	70.00	24.24	60.19	3.68
	R	36	1.94	54.00	49.09	46.02	3.44
	FS	38	2.09	45.50	8.37	51.52	3.86
3	HC	20	1.13	27.00	13.15	32.92	3.11
	LC	18	1.07	20.00	5.29	21.10	2.17
	PA	22	1.14	43.00	NaN	30.78	2.94
	R	18	1.65	18.00	0.73	18.32	2.08
	FS	19	1.89	19.33	1.87	20.71	2.14
4	HC	17	1.14	18.67	2.96	25.99	3.34
	LC	24	1.75	25.50	3.49	26.78	2.60
	PA	24	1.54	42.00	49.09	40.27	3.36
	R	7	1.47	10.00	NaN	9.97	1.70
	FS	7	1.46	7.50	3.74	8.44	1.32

Main diversity indexes calculated at family taxonomy rank for each sample/fraction.
