# Supplementary material for: The Active Human Gut Microbiota Differs from the Total Microbiota
Source: PLoS One. 2011 Jul 28;6(7):e22448. doi: 10.1371/journal.pone.0022448 (PMC3145646; doi:10.1371/journal.pone.0022448)
Supplement: Table S2 — Probes used in this work [69] –[73] . (PDF) [file pone.0022448.s010.pdf]

Probe	Phylogroup	Sequence	Reference
LGC354B	Firmicutes	CGGAAGATTCCCTACTGC	[69]
Erec482	Clostridium coccoides-Eubacterium rectale group	GCTTCTTAGTCARGTACCG	[70]
Fprau645	Firmicutes	CCTCTGCACTACTCAAGAAAAAC	[71]
CFB560	Subgroup of Bacteroidetes	WCCCTTTAAACCCART	[72]
CFB719	Classes of Bacteroidetes, Flavobacteria Sphingobacteria	AGCTGCCTTCGCAATCGG	[73]
