# Supplementary material for: The Active Human Gut Microbiota Differs from the Total Microbiota
Source: PLoS One. 2011 Jul 28;6(7):e22448. doi: 10.1371/journal.pone.0022448 (PMC3145646; doi:10.1371/journal.pone.0022448)
Supplement: Table S3 — Multiplex Identifiers (MIDs) list and universal 16S rRNA primers used in this work [61] . (PDF) [file pone.0022448.s011.pdf]

MID	Sequence
MID1	TAACCAACC
MID2	TAACCGGAA
MID3	TAACCTTCC
MID4	TAAGCCGAA
MID5	TAAGCGGAT
MID6	TAAGGTTCC
MID7	TAATACGCC
MID8	TACACCACA
MID9	TACATCTCT
MID10	TACAGACTC
MID56	TGAAGTGCA
MID57	TGACAGAGT
MID58	TGAGATGTG
MID59	TGAGTGACA
MID60	TGATCTGCA
MID61	TGCAACGAA
MID62	TGCATAATG
MID63	TGCCGTAAT
MID64	TGCGGTTAT
MID65	TGCTACCAA

Finalstructure.

Forward:
MID-WGNGNNGW(Permutag)-TC(linker)-AGAGTTTGATCMTGGCTCAG(ForwardPrimer [61])

Reverse:
MID-WGNGNNGW(Permutag)-TC(linker)-CCGCGGCKGCTGGCAC(ReversePrimer [61])
